# A Facile Design for Water‐Oxidation Molecular Catalysts Precise Assembling on Photoanodes

**DOI:** 10.1002/advs.202305919

**Published:** 2023-11-20

**Authors:** Wenchao Jiang, Siyuan Li, Qi Sui, Yujie Gao, Fei Li, Lixin Xia, Yi Jiang

**Affiliations:** ^1^ College of Chemistry Liaoning University Shenyang Liaoning 110036 China; ^2^ School of Chemical and Materials Science University of Science and Technology of China Hefei Anhui 230026 China; ^3^ State Key Laboratory of Fine Chemicals Dalian University of Technology Dalian Liaoning 116024 China; ^4^ Yingkou Institute of Technology Yingkou Liaoning 115100 China

**Keywords:** bismuth vanadate, hole transfer channels, molecular catalysts, photoelectrochemical water oxidation

## Abstract

Regulating the interfacial charge transfer behavior between cocatalysts and semiconductors remains a critical challenge for attaining efficient photoelectrochemical water oxidation reactions. Herein, using bismuth vanadate (BiVO_4_) photoanode as a model, it introduces an Au binding bridge as holes transfer channels onto the surfaces of BiVO_4_, and the cyano‐functionalized cobalt cubane (Co_4_O_4_) molecules are preferentially immobilized on the Au bridge due to the strong adsorption of cyano groups with Au nanoparticles. This orchestrated arrangement facilitates the seamless transfer of photogenerated holes from BiVO_4_ to Co_4_O_4_ molecules, forming an orderly charge transfer pathway connecting the light‐absorbing layer to reactive sites. An exciting photocurrent density of 5.06 mA cm^−2^ at 1.23 V versus the reversible hydrogen electrode (3.4 times that of BiVO_4_) is obtained by the Co_4_O_4_@Au(A)/BiVO_4_ photoanode, where the surface charge recombination is almost completely suppressed accompanied by a surface charge transfer efficiency over 95%. This work represents a promising strategy for accelerating interfacial charge transfer and achieving efficient photoelectrochemical water oxidation reaction.

## Graphical abstract

The BiVO_4_ photoanode's surface is adorned with assemblies of Au@Co_4_O_4_ aggregates, which consist of cyano‐functionalized Co_4_O_4_ molecules and Au nanoparticles. This distinctive arrangement results in the remarkable performance of the Au@Co_4_O_4_(A)/BiVO_4_ photoanode, which attains an impressive photocurrent density of 5.06 mA cm^−2^ and near‐unit charge‐transfer efficiency at 1.23 V vs. RHE.

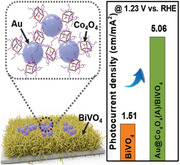

## 1 Introduction

Artificial photosynthesis, converting solar energy into chemical energy, presents a promising avenue for sustainable fuel production.^[^ ^1^ ^]^ The seminal work by Fujishima and Honda demonstrated water splitting on a TiO_2_ photoanode, heralding the exploration of photoelectrochemical (PEC) devices based on light‐absorbing metal oxide semiconductors.^[^ ^2^ ^]^ Within a PEC system, the photoanode holds paramount significance as the site governing the oxygen evolution reaction (OER), which entails intricate proton‐coupled electron transfer steps.^[^ ^3^ ^]^ To emulate the roles of the P680 chromophore and the oxygen‐evolving Mn_4_CaO_5_ cluster in natural Photosystem II (PSII), an adeptly designed photoanode typically integrates a light‐harvesting semiconductor antenna and a water oxidation catalyst (WOC) for surface catalytic OER.^[^ ^4^ ^]^ Nevertheless, the prevailing approach involves combining metal oxide cocatalysts with semiconductors to form solid‐state materials, inadvertently constraining contact areas between cocatalyst and reactants, as well as the availability of active catalytic sites.^[^ ^5^ ^]^ In particular, the solid‐solid interface between nanoparticle cocatalysts and semiconductors exacerbates the transport distance of photogenerated charges, leading to pronounced photogenerated charge recombination.

In contrast to inorganic solid cocatalysts, molecular cocatalysts offer the advantage of facile modulation through substitution. Transition metal complexes, particularly cobalt cubane molecules (Co_4_O_4_), sharing structural similarities with the oxygen‐evolving complex Mn_4_CaO_5_ in PSII of green plants, were initially reported by Christou and colleagues.^[^ ^6^ ^]^ Co_4_O_4_ molecules have emerged as highly efficient water oxidation cocatalysts in homogeneous solutions. However, their performance tends to be unsatisfactory when integrated onto semiconductor surfaces, primarily due to poor interfacial charge transfer and inadequate surface anchoring.^[^ ^7^ ^]^ In natural PSII, numerous amino acids and coenzymes, in addition to the chromophore and OER center, regulate the direction of charge separation and transfer.^[^ ^8^ ^]^ Drawing inspiration from this, charge transfer mediators, such as graphene, have demonstrated the ability to facilitate hole transfer from semiconductors to cocatalysts.^[^ ^9^ ^]^ Nevertheless, the transfer of photogenerated charges between layers remains suboptimal due to the lack of a specific transfer channel that establishes a robust bond between the semiconductor and cocatalysts. Thus, the development of a charge transfer mediator is imperative, one that can not only govern the charge transfer pathway of semiconductors but also effectively bind with cocatalysts to promote charge transfer.

In this study, we present an artificial photoanode comprising a BiVO_4_ semiconductor as the light absorber, Co_4_O_4_ molecules as the cocatalyst, and Au nanoparticles (NPs) as the binding bridge. The integration of Au NPs serves to accumulate photogenerated holes, effectively operating as “hole transfer channels” at the BiVO_4_ surface. The terminal catalytic site, represented by Co_4_O_4_ molecular cocatalysts, is meticulously affixed to the Au surface via cyano anchoring groups. This rational arrangement establishes an ordered pathway for charge transfer between BiVO_4_ and Co_4_O_4_ molecules. Diverging from conventional “molecular cocatalyst/semiconductor” binary photoanodes, the systematically assembled photoanode, characterized by the “Au@Co_4_O_4_” structure, facilitates directional hole transfer along the Au channels, considerably mitigating surface charge recombination. As a result, the Au@Co_4_O_4_(A)/BiVO_4_ photoanode showcases remarkable performance in PEC water splitting. It attains an impressive photocurrent density of 5.06 mA cm^−2^ (3.4 times that of pristine BiVO_4_) at 1.23 V versus the reversible hydrogen electrode (RHE), accompanied by an ultrahigh surface charge transfer efficiency exceeding 95%.

## 2 Results and Discussion

Despite being proposed as efficient WOC for BiVO_4_ photoanode, Co_4_O_4_ molecules often face performance limitations due to inadequate interfacial charge transfer and surface anchoring when integrated onto BiVO_4_ surface. Au NPs, previously investigated extensively as charge transfer mediators through interfacing with semiconductors or cocatalysts, offer potential solutions in this context.^[^ ^10^ ^]^ In this study, we leverage Au NPs as charge transfer channels, specifically aiming to achieve selective assembly of Co_4_O_4_ molecules on their surfaces. This selective assembly is a critical stride toward enabling efficient photogenerated hole transfer from BiVO_4_ to Co_4_O_4_ molecules, thereby enhancing the overall performance of the PEC water oxidation process.

### 2.1 Interaction of Au NPs and Co_4_O_4_ Molecules

**Figure 1:**
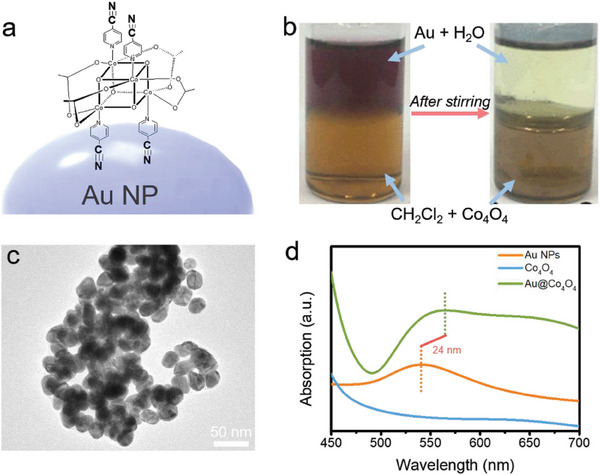
a) Schematic illustration of the structure of Au@Co_4_O_4_. b) The biphasic adsorption experiment of Au sol and Co_4_O_4_ molecules. c) Transmission electron microscope (TEM image of Au@Co_4_O_4_ aggregates. d) UV–vis spectra of Au NPs, Co_4_O_4_ molecules and Au@Co_4_O_4_. aggregates.

In this study, cyano‐functionalized Co_4_O_4_ molecules were selected for their ability to establish robust coordination bonds with heavy metals like Au, owing to the cyano groups they bear.^[^ ^11^ ^]^ Consequently, Co_4_O_4_ molecules readily adsorb onto the Au surface, creating an organic–inorganic hybrid nanostructure termed Au@Co_4_O_4_ (**Figure** **1**a). The presence of characteristic Raman vibrations within the range of 1000–2300 cm^−1^, attributed to the pyridine ring and cyano group, validates the successful synthesis of cyano‐functionalized Co_4_O_4_ molecules (Figure S1, Supporting Information). Leveraging steric and geometric attributes, individual Co_4_O_4_ molecule serves as bridges, linking two Au NPs together to form Au@Co_4_O_4_ aggregate. A biphasic adsorption experiment corroborates this aggregation phenomenon.^[^ ^12^ ^]^ As depicted in Figure 1b, citrate‐encapsulated Au NPs disperse in the upper water phase, while the lower dichloromethane layer contains Co_4_O_4_ molecules. Over time, stirring prompts the gradual gathering of Au NPs at the interface, resulting in the upper layer becoming colorless and transparent. Furthermore, introducing Co_4_O_4_ molecules into an Au colloid solution leads to noticeable agglomeration of Au NPs (Figure 1c; Figure S2, Supporting Information). Spectral confirmation of this aggregation is provided by the UV–vis spectrum, which exhibits a substantial red shift of 24 nm upon Co_4_O_4_ molecules and Au NPs co‐mingling (Figure 1d). These collective outcomes denote a closely packed interfacial arrangement between Co_4_O_4_ molecules and Au NPs, thereby offering ample potential for subsequent integration.

### 2.2 Fabrication and Characterization of BiVO_4_‐Based Photoanodes

**Figure 2:**
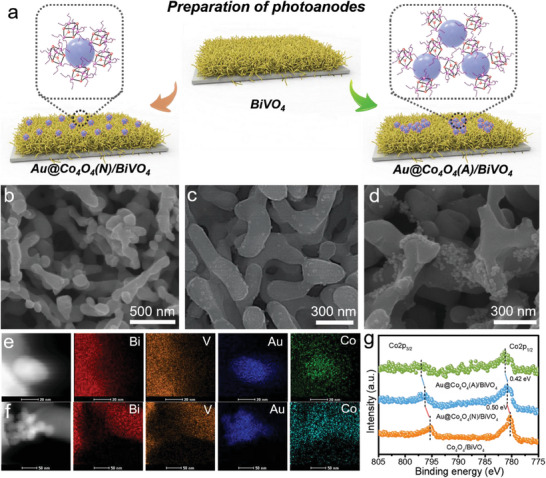
a) The preparation routes of Au@Co_4_O_4_(N)/BiVO_4_ and Au@Co_4_O_4_(A)/BiVO_4_ photoanodes. SEM images of b) BiVO_4_, c) Au@Co_4_O_4_(N)/BiVO_4_ and d) Au@Co_4_O_4_(A)/BiVO_4_ photoanodes. HAADF‐STEM and EDS elemental mapping images of e) Au@Co_4_O_4_(N)/BiVO_4_ and f) Au@Co_4_O_4_(A)/BiVO_4_ photoanodes. g) High‐resolution XPS of Co 2p spectra for BiVO_4_‐based photoanodes.

Two strategies were employed for the photoanode assembly, as illustrated in **Figure** **2**a. In Route 1, Au NPs were initially deposited onto the BiVO_4_ surface, followed by the introduction of Co_4_O_4_ molecules onto the Au/BiVO_4_ photoanode to yield Au@Co_4_O_4_ NPs (designated as Au@Co_4_O_4_(N)/BiVO_4_). Alternatively, Route 2 involved blending Co_4_O_4_ molecules and Au NPs to create Au@Co_4_O_4_ aggregates, which were subsequently directly integrated onto the BiVO_4_ film (designated as Au@Co_4_O_4_(A)/BiVO_4_). The porous nature of the BiVO_4_ films provided ample loading sites for the catalysts (Figure 2b). Loading Co_4_O_4_ molecules onto the Au/BiVO_4_ photoanode had minimal impact on the morphology of the Au NPs, owing to their monolayer deposition (Figure 2c; Figure S3, Supporting Information). In contrast, the Scanning electron microscope (SEM) image of Au@Co_4_O_4_(A)/BiVO_4_ distinctly revealed the presence of Au@Co_4_O_4_ aggregates on the photoanode surface (Figure 2d).

To validate the distribution of the Co element on Au@Co_4_O_4_(N)/BiVO_4_ and Au@Co_4_O_4_(A)/BiVO_4_ photoanodes, high‐angle annular dark field scanning transmission electron microscopy (HAADF‐STEM) and energy‐dispersive X‐ray spectroscopy (EDS) elemental mapping were performed (Figure 2e,f). The results indicated that the majority of Co elements were situated around the Au NPs in both the Au@Co_4_O_4_(N)/BiVO_4_ and Au@Co_4_O_4_(A)/BiVO_4_ photoanodes, suggesting preferential anchoring of Co_4_O_4_ molecules to the Au surface. The electronic coupling interaction within the Au@Co_4_O_4_ NPs and aggregates was verified using X‐ray photoelectron spectroscopy (XPS). The Co 2p binding energies for Au@Co_4_O_4_(N)/BiVO_4_ and Au@Co_4_O_4_(A)/BiVO_4_ exhibited noticeable positive shifts compared to Co_4_O_4_/BiVO_4_ (Figure 2g), indicating enhanced electron transfer from Co_4_O_4_ molecules to BiVO_4_ facilitated by the presence of Au NPs. Apart from the typical peak of BiVO_4_ at 826 cm^−1^, the characteristic vibrations of pyridine ring in Co_4_O_4_ molecules between 1000 and 1800 cm^−1^ were found in the Raman spectra of Au@Co_4_O_4_(N)/BiVO_4_ and Au@Co_4_O_4_(A)/BiVO_4_. In particular, a characteristic peak at 2237 cm^−1^ corresponds to the vibration of cyano group in Co_4_O_4_ molecules, appears in the Raman spectra of Au@Co_4_O_4_(N)/BiVO_4_ and Au@Co_4_O_4_(A)/BiVO_4_ (Figure S1, Supporting Information). Furthermore, UV–vis absorption spectra of the photoanodes incorporating Au NPs exhibited a distinct absorption peak ≈540 nm, attributed to plasmonic light absorption induced by Au NPs. Notably, the introduction of Co_4_O_4_ molecules had negligible impact on the light absorption characteristics of the photoanodes (Figure S4, Supporting Information).

### 2.3 Photoelectrochemical (PEC) Water Oxidation Over BiVO_4_‐Based Photoanodes

**Figure 3:**
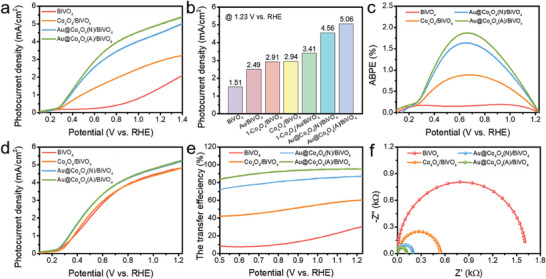
The LSV curves a) and the photocurrent density value at 1.23 V b) of BiVO_4_‐based photoanodes. c) The ABPE values, d) LSV curves in Na_2_SO_3_ solution, e) surface charge transfer efficiencies, and f) EIS data of BiVO_4_‐based photoanodes. (The illuminated area of photoanodes is 1 cm^2^).

The PEC performance of the fabricated photoanodes was assessed within a typical three‐electrode cell under AM 1.5G illumination in a neutral phosphate buffer solution. As depicted in **Figure** **3**a, the unmodified BiVO_4_ photoanode displayed a modest photocurrent density of 1.51 mA cm^−2^ at 1.23 V versus RHE. Upon incorporation of Co_4_O_4_ molecules, a promising enhancement in photocurrent density was evident for the Co_4_O_4_/BiVO_4_ configuration. Particularly noteworthy, both Au@Co_4_O_4_(N)/BiVO_4_ and Au@Co_4_O_4_(A)/BiVO_4_ exhibited further augmented photocurrent density when Co_4_O_4_ molecules were anchored onto the BiVO_4_ surface via Au mediators, yielding photocurrent density of 4.65 mA cm^−2^ and 5.06 mA cm^−2^ at 1.23 V versus RHE, respectively, surpassing those reported in previous studies (Table S2, Supporting Information). Furthermore, the onset potential for both Au@Co_4_O_4_(N)/BiVO_4_ and Au@Co_4_O_4_(A)/BiVO_4_, corresponding to a photocurrent density of 0.5 mA cm^−2^, was cathodically shifted by ≈550 mV compared to pristine BiVO_4_. While Au NPs and Co_4_O_4_ molecules serve as cocatalysts that collaborate in catalyzing the water oxidation reaction, it's crucial to emphasize that the modest increase in photocurrent density observed in Au/BiVO_4_ compared to BiVO_4_ can be primarily attributed to the effective function of Au NPs as “hole transfer channels” (Figures S5–S7, Supporting Information).

To enhance the vertical integration of the Au@Co_4_O_4_ hybrid nanostructure, cobalt cubane molecules (1‐Co_4_O_4_) without functional groups were also introduced onto the Au/BiVO_4_ surface for comparative analysis (Figures S8 and S9, Supporting Information). Notably, both 1‐Co_4_O_4_/BiVO_4_ and Co_4_O_4_/BiVO_4_ configurations exhibited nearly identical photocurrent density. However, a distinct trend emerged in the photocurrent behavior of Au‐incorporated photoanodes (Au@Co_4_O_4_(A)/BiVO_4_ > Au@Co_4_O_4_(N)/BiVO_4_ > 1‐Co_4_O_4_/Au/BiVO_4_) in accordance with the interaction affinity between the Co_4_O_4_ molecules and Au NPs (Figure 3b). This observation underscores the critical influence of their binding mode on interfacial charge transfer. Evaluation of linear sweep voltammograms facilitated the calculation of applied bias photon‐to‐current efficiency (ABPE) values for the integrated photoanodes, as depicted in Figure 3c. The highest ABPE values were attained by the Au@Co_4_O_4_(N)/BiVO_4_ and Au@Co_4_O_4_(A)/BiVO_4_ photoanodes, reaching 1.64% at 0.65 V and 1.88% at 0.66 V, respectively.

### 2.4 The Surface Charge Transfer and Recombination of BiVO_4_‐Based Photoanodes

To evaluate the charge transfer efficiencies of the BiVO_4_‐based photoanodes, the hole scavenger Na_2_SO_3_ was employed to alleviate surface charge recombination.^[^ ^13^ ^]^ As illustrated in Figure 3d, all photoanodes incorporating Au NPs displayed comparable photocurrent levels, surpassing those of pristine BiVO_4_ in Na_2_SO_3_ solution, which suggest an indication of enhanced hole generation upon Au NPs integration. Surface charge transfer efficiencies (*η*_trans_) of the BiVO_4_‐based photoanodes were calculated using the formula *η*_trans_ = *J*_H2O_/*J*_Na2SO3_, with *J*_H2O_ and *J*_Na2SO3_ denoting photocurrent density measured with and without Na_2_SO_3_, respectively. The data in Figure 3e reveals that Co_4_O_4_/BiVO_4_ exhibited higher *η*_trans_ than BiVO_4_. Nevertheless, the values remained below 60% across the voltage range, underscoring the constrained charge transfer at the Co_4_O_4_/BiVO_4_ photoanode interface. Remarkably, immobilizing Co_4_O_4_ molecules onto the BiVO_4_ photoanode surface via an Au mediator led to a successive increase in *η*_trans_ for the resultant Au@Co_4_O_4_(N)/BiVO_4_ and Au@Co_4_O_4_(A)/BiVO_4_ photoanodes, aligning with the photocurrent density order depicted in Figure 3a. Notably, Au@Co_4_O_4_(A)/BiVO_4_ exhibited an exceptional *η*_trans_, reaching a maximum value of 95.2% at 1.23 V versus RHE. Electrochemical impedance spectroscopy (EIS) measurements of the prepared photoanodes were consistent with the charge transfer efficiency results (Figure 3f). The semidiameters of the semicircles in the Nyquist plots for BiVO_4_, Co_4_O_4_/BiVO_4_, Au@Co_4_O_4_(N)/BiVO_4_, and Au@Co_4_O_4_(A)/BiVO_4_ followed a sequence of increasing to decreasing order. This sequence signifies a corresponding acceleration in interfacial charge transfer.

**Figure 4:**
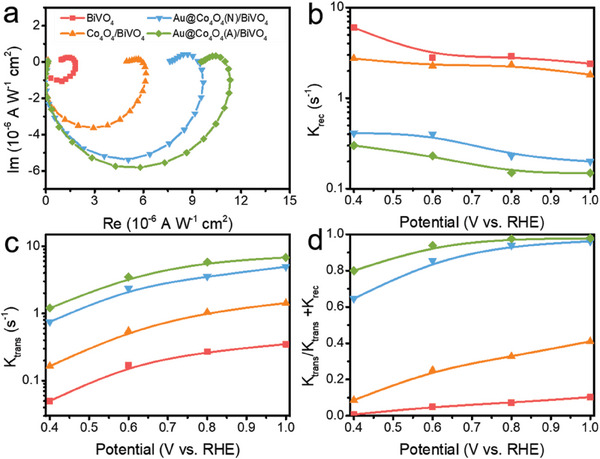
a) IMPS responses at 0.8 V versus RHE of BiVO_4_‐based photoanodes under 460 nm. b) The charge recombination rate constants (*k*_trans_), c) charge transfer rate constants (*k*_rec_), and d) charge transfer efficiencies of BiVO_4_‐based photoanodes obtained from IMPS analysis.

To more accurately assess the charge transfer and charge recombination kinetics, we conducted intensity‐modulated photocurrent spectroscopy (IMPS) utilizing monochromatic light at 460 nm.^[^ ^14^ ^]^ **Figure** **4**a presents typical IMPS responses of BiVO_4_‐based photoanodes at 0.8 V versus RHE, which unveil insights into charge transfer and recombination kinetics. By analyzing the semicircles in the IMPS plots (Figure S10, Supporting Information), the charge transfer rate constant (*k*_trans_) and charge recombination rate constant (*k*_rec_) were derived at various applied potentials. Figure 4b,c illustrate that both Au@Co_4_O_4_(N)/BiVO_4_ and Au@Co_4_O_4_(A)/BiVO_4_ photoanodes exhibit lower *k*_rec_ but elevated *k*_trans_ values across the entire voltage spectrum compared to Co_4_O_4_/BiVO_4_ photoanodes. These findings underscore that the introduction of Au NPs between BiVO_4_ and Co_4_O_4_ molecules can effectively curtail interfacial charge recombination while accelerating charge transfer. Determination of the steady‐state charge transfer efficiency (TE) for each photoanode involved calculating *k*_trans_/(*k*_trans_ + *k*_rec_). Particularly noteworthy, IMPS analysis revealed that the highest TE value for Au@Co_4_O_4_(A)/BiVO_4_ was 97.2% at 1.0 V (Figure 4d), consistent with the computed outcome in Figure 3e. This nearly perfect efficiency underscores the pivotal role played by Au NPs in enhancing interfacial charge transfer.

### 2.5 Identification of Au NPs as Hole Transfer Channels

**Figure 5:**
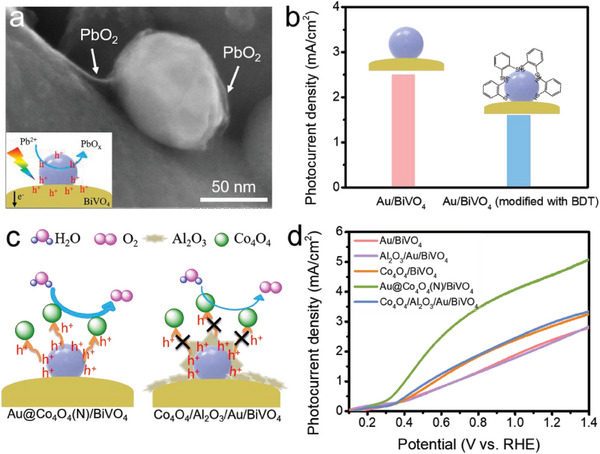
a) SEM image of Au/BiVO_4_ photoanode after hole‐involved oxidative photo‐deposition of PbO_2_ from Pb^2+^ ion. b) The photocurrent density values comparison of Au/BiVO_4_ with or without the treatment of BDT at 1.23 V. c) Schematic illustration of the water oxidation mechanism comparison with or without the introduction of Al_2_O_3_ interlayer. d) LSV curves of BiVO_4_‐based photoanodes.

To establish the crucial function of Au NPs as hole transfer channels on the BiVO_4_ surface, we undertook targeted control experiments and characterizations. Initially, in situ photochemical deposition was employed to examine the sites of oxidation reactions on the Au/BiVO_4_ photoanode.^[^ ^15^ ^]^ We observed that the hole‐mediated oxidative deposition of PbO_2_ from Pb^2+^ was notably concentrated around the Au NPs, indicative of photogenerated hole accumulation near Au NPs (**Figure** **5**a). This observation supports the notion that the Au NPs can serve as hole trappers, effectively capturing photogenerated holes from BiVO_4_. Additionally, we conducted an experiment involving treatment of the Au/BiVO_4_ photoanode with 1,2‐benzenedithiol (BDT). This treatment selectively forms Au─S covalent bonds to coat the surface of Au NPs. Notably, this treatment hindered water molecules from interacting with the Au NPs, while the PEC activity of BiVO_4_ remained unaffected (Figure S11, Supporting Information). As depicted in Figure 5b, following the BDT treatment, the Au channels became obstructed, resulting in a substantial decrease in the photocurrent density of Au/BiVO_4_, approaching that of pristine BiVO_4_. This outcome conclusively validates the pivotal role of Au NPs as effective hole transfer channels.

To delve deeper into the significance of charge transfer between Au NPs and Co_4_O_4_ molecules, an Al_2_O_3_ film was introduced onto the surface of the Au/BiVO_4_ photoanode.^[^ ^16^ ^]^ The Al_2_O_3_ film, with a thickness of ≈3.5 nm, provided complete coverage of the Au/BiVO_4_ surface (Figure S12, Supporting Information). Serving as an interlayer, this film effectively separated the Co_4_O_4_ molecules from the Au NPs, preventing Co_4_O_4_ molecules attachment to the Au surface and the formation of the Au@Co_4_O_4_ hybrids structure (Figure 5c). Notably, the photocurrent density of Al_2_O_3_/Au/BiVO_4_ remained comparable to that of Au/BiVO_4_, indicating that the presence of the Al_2_O_3_ film did not disrupt charge transfer at the Au/BiVO_4_ surface. However, upon the introduction of Co_4_O_4_ molecules, the resulting Co_4_O_4_/Al_2_O_3_/Au/BiVO_4_ photoanode exhibited a marked reduction in activity compared to Au@Co_4_O_4_(N)/BiVO_4_ (Figure 5d). This observation underscores the essential role of charge transfer between Au NPs and Co_4_O_4_ molecules in ensuring the efficient PEC performance of the photoanode.

To reinforce this hypothesis, we introduced 1‐Co_4_O_4_ molecules lacking cyano groups for comparative analysis. Notably, these 1‐Co_4_O_4_ molecules were unable to assemble on the surface of Au NPs, as evidenced in Figure S13 (Supporting Information). Direct introduction of 1‐Co_4_O_4_ molecules onto the Au/BiVO_4_ surface resulted in only a marginal increase in photocurrent density, indicative of the absence of an effective charge transfer channel between BiVO_4_ and 1‐Co_4_O_4_ molecules. Furthermore, the introduction of an Al_2_O_3_ film onto the Au/BiVO_4_ photoanode had no discernible impact on the photocurrent density of 1‐Co_4_O_4_/Al_2_O_3_/Au/BiVO_4_ and 1‐Co_4_O_4_/Au/BiVO_4_ at 1.23 V versus RHE (Figure S14, Supporting Information). Based on the aforementioned findings, we posit that the enhanced charge transfer between Co_4_O_4_ molecules and BiVO_4_ facilitated by the Au bridge significantly contributes to the augmentation of PEC water oxidation.

**Figure 6:**
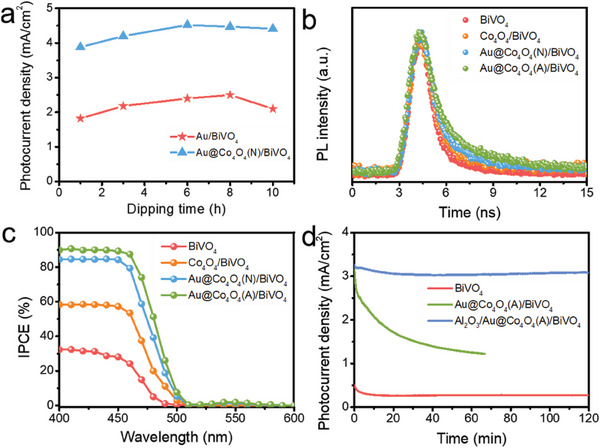
a) Photocurrent densities of Au/BiVO_4_ and Au@Co_4_O_4_(N)/BiVO_4_ photoanodes with different Au NPs loading at 1.23 V. b) PL decay profiles at 490 nm detection of BiVO_4_‐based photoanodes under 460 nm excitation. c) IPCE values of BiVO_4_‐based photoanodes at 1.23 V. d) I–t curves of BiVO_4_‐based photoanodes under 0.7 V.

Of significance is the observation that the photocurrent density of Au@Co_4_O_4_(A)/BiVO_4_, where Co_4_O_4_ molecules and Au NPs form Au@Co_4_O_4_ aggregates, surpasses that of Au@Co_4_O_4_(N)/BiVO_4_, where Co_4_O_4_ molecules and Au NPs form Au@Co_4_O_4_ NPs. To eliminate the possibility that the improved PEC performance observed in Au@Co_4_O_4_(A)/BiVO_4_ was solely due to an increased loading of Au NPs, we prepared a series of photoanodes with varying amounts of Au NPs by adjusting the immersion time. As the loading of Au NPs increases, the resulting photoanodes exhibit a characteristic volcano‐shaped curve with respect to photocurrent density, where excessive Au NPs loading leads to a decline in PEC activity (**Figure** **6**a; Figure S15, Supporting Information). Numerical simulations utilizing finite‐difference time‐domain (FDTD) for plasmon‐induced electric field intensity reveal that Au NPs aggregations generate enhanced regions within the nanogap, outperforming isolated Au NPs (Figure S16, Supporting Information).^[^ ^17^ ^]^ In the Au@Co_4_O_4_ aggregates, Co_4_O_4_ molecules are strategically positioned at the nanogap of Au NPs, acting as linkers. This arrangement maximizes the localized near‐field enhancement, fostering hole accumulation, and consequently leading to improved OER catalysis.

To gain deeper insights into the impact of multicomponent photoanodes on interface modulation, we assessed the photovoltage and surface recombination rate by conducting open circuit voltage decay measurements on the photoanodes.^[^ ^18^ ^]^ The sequence of photovoltages and recovery constants “b” for the photoanodes aligns as Au@Co_4_O_4_(A)/BiVO_4_ > Au@Co_4_O_4_(N)/BiVO_4_ > Co_4_O_4_/BiVO_4_ > BiVO_4_, as anticipated. This corresponds to a progressive reduction in the driving force and a subsequent increase in charge recombination (Figure S17, Supporting Information). We also employed time‐resolved photoluminescence (TRPL) to determine the carrier lifetime (Figure 6b).^[^ ^19^ ^]^ The PL decay curves were fitted using a biexponential function, enabling the capture of the initial rapid decay (*τ*_1_) and the subsequent slower decay (*τ*_2_), where *τ*_1_ signifies the fall of photogenerated electrons near the conduction band, and *τ*_2_ indicates the recombination of electron‐hole pairs. The average carrier lifetimes of Au@Co_4_O_4_(A)/BiVO_4_ (6.8 ns), Au@Co_4_O_4_(N)/BiVO_4_ (4.0 ns), Co_4_O_4_/BiVO_4_ (2.3 ns), and BiVO_4_ (1.8 ns) were determined using the formula *τ*_ave_ = *P*_1_ *τ*_1_ + *P*_2_ *τ*_2_ (Table S1, Supporting Information). These results unequivocally affirm the pivotal role played by Au NPs in facilitating efficient hole transfer from BiVO_4_ to Co_4_O_4_ molecules.

Figure 6c illustrates the incident photon‐to‐current conversion efficiencies (IPCEs) of the sample photoanodes. Notably, the IPCE values of Au@Co_4_O_4_(N)/BiVO_4_ and Au@Co_4_O_4_(A)/BiVO_4_ exhibit substantial enhancements compared to those of Co_4_O_4_/BiVO_4_ and BiVO_4_ across the entire wavelength range. A discrete rise in IPCE ≈540 nm for Au@Co_4_O_4_(N)/BiVO_4_ and Au@Co_4_O_4_(A)/BiVO_4_ can be attributed to plasmon‐induced water oxidation. The integrated IPCE curves over the AM 1.5G solar spectrum yield photocurrent densities of 4.51 mA cm^−2^ and 5.01 mA cm^−2^ at 1.23 V for Au@Co_4_O_4_(N)/BiVO_4_ and Au@Co_4_O_4_(A)/BiVO_4_ photoanodes, respectively (Figure S18, Supporting Information), aligning closely with the experimentally measured photocurrent densities.

Stability is a pivotal concern for PEC devices, including the photoanode. Au‐decorated semiconductor photoanodes, as a rule, exhibit unsatisfactory stability.^[^ ^20^ ^]^ Similarly, while Au@Co_4_O_4_(A)/BiVO_4_ demonstrates remarkably high PEC activity, a discernible decline in photocurrent density during constant‐potential electrolysis is evident. Addressing this challenge, we introduced an Al_2_O_3_ protective layer to the outer surface of the Au@Co_4_O_4_(A)/BiVO_4_ photoanode through atomic layer deposition (ALD).^[^ ^21^ ^]^ With the protective Al_2_O_3_ layer, the resulting Al_2_O_3_/Au@Co_4_O_4_(A)/BiVO_4_ photoanode maintains a photocurrent density comparable to Au@Co_4_O_4_(A)/BiVO_4_ while exhibiting significantly enhanced stability (Figure S19, Supporting Information). Remarkably, over 95% of the initial photocurrent density is retained after 120 min of photoelectrolysis (Figure 6d). The faradaic efficiency of the Al_2_O_3_/Au@Co_4_O_4_(A)/BiVO_4_ photoanode for water oxidation is ≈88.9%, strongly indicating that the photocurrent originates from the water oxidation reaction (Figure S20, Supporting Information). Further investigations into achieving more stable photoanodes through adjustments in protective layer type and thickness are currently underway in our laboratory.

## 3 Conclusion

In summary, our study presents a novel multicomponent photoanode designed to modulate interfacial charge transfer behavior. We introduce Au NPs as efficient hole transfer channels, facilitating the transfer of photogenerated charges between the BiVO_4_ light absorber and Co_4_O_4_ molecular cocatalysts. Notably, Co_4_O_4_ molecules and Au NPs are meticulously assembled onto the photoanode surface through cyano groups, resulting in the formation of unique Au@Co_4_O_4_ nanoparticles and aggregates. This distinctive structural arrangement effectively suppresses surface charge recombination and significantly accelerates water oxidation kinetics. Among the designed photoanodes, the Au@Co_4_O_4_(A)/BiVO_4_ configuration demonstrates the highest activity, yielding a substantial photocurrent density of 5.06 mA cm^−2^ at 1.23 V. Furthermore, an impressive near‐unit charge transfer efficiency and an enhanced ABPE value of 1.88% at 0.66 V (9.7 times that of BiVO_4_) are achieved. Supported by control experiments and an analysis of carrier transfer kinetics, these enhancements in PEC performance are primarily attributed to the hole channel effect of Au NPs and the facilitated interfacial charge transfer. These findings underscore the critical significance of precisely regulating hole transfer between semiconductors and molecular cocatalysts, providing a viable strategy for developing efficient hybrid photoanodes for PEC solar conversion.

## Conflict of Interest

The authors declare no conflict of interest.

## Supporting information

Supporting Information

## Data Availability

The data that support the findings of this study are available from the corresponding author upon reasonable request.;
