# Supplementary material for: A Facile Design for Water‐Oxidation Molecular Catalysts Precise Assembling on Photoanodes
Source: Adv Sci (Weinh). 2023 Nov 20;11(2):2305919. doi: 10.1002/advs.202305919 (PMC10787085; doi:10.1002/advs.202305919)
Supplement: Supplementary file 1 — Supporting Information [file ADVS-11-2305919-s001.pdf]

ADVANCED SCIENCE
Open Access

Supporting Information

for *Adv. Sci.*, DOI 10.1002/advs.202305919

A Facile Design for Water-Oxidation Molecular Catalysts Precise Assembling on Photoanodes

Wenchao Jiang, Siyuan Li, Qi Sui, Yujie Gao, Fei Li, Lixin Xia* and Yi Jiang**

Supporting Information

A Facile Design for Water-Oxidation Molecular Catalysts Precise Assembling on Photoanodes

Wenchao Jiang, Siyuan Li, Qi Sui, Yujie Gao, Fei Li[*]*, Lixin Xia*[*]*, and Yi Jiang*[*]

W. Jiang, S. Li, Q. Sui, Y. Gao, L. Xia,[*] Y. Jiang[*]

College of Chemistry, Liaoning University, Shenyang 110036, Liaoning, China

E-mail: lixinxia@lnu.edu.cn; jiangyi@lnu.edu.cn

F. Li[*]

State Key Laboratory of Fine Chemicals, Dalian University of Technology, Dalian 116024, Liaoning, China

E-mail: lifei@dlut.edu.cn

L. Xia[*]

Yingkou Institute of Technology, Yingkou 115100, Liaoning, China

W. Jiang

School of Chemical and Materials Science, University of Science and Technology of China, Hefei 230026, Anhui, China

Experimental Section

Synthesis of $Co_4O_4(O_2CMe)_4(4\text{-}CNpy)_4$ (Co_4O_4) molecular catalyst. The preparation of Co_4O_4 was conducted using the following method: $Co(NO_3)_2{\cdot}6H_2O$ (2.90 g, 10 mmol) and $CH_3COONa{\cdot}3H_2O$ (2.70 g, 10 mmol) were initially dissolved in a 250 mL two-necked flask containing 30 mL of methanol. The solution was then heated to reflux temperature. Subsequently, 10 mmol of pyridine (4-

Cyanopyridine) and 5 mL of hydrogen peroxide (30%) were separately introduced into the reaction mixture, followed by stirring for a duration of 4 hours at reflux temperature. After completion, the cooled reaction mixture was subjected to concentration using a rotary evaporator, with the resulting aqueous layer being separated by adding CH_2Cl_2. The light-pink aqueous layer was discarded, and the CH_2Cl_2 layer was concentrated using a rotary evaporator. Finally, the addition of petroleum ether to the CH_2Cl_2 solution yielded the yellow-green product. The obtained product was further dried under vacuum conditions in a desiccator (72% based on cobalt).

Fabrication of $BiVO_4$ photoanode. The fabrication of the $BiVO_4$ photoanode followed a well-established procedure. Initially, 2.91 g of $Bi(NO_3)_3\cdot 5H_2O$ was dissolved in 150 mL of deionized water containing 9.96 g of KI, and the pH of the solution was subsequently adjusted to 1.7 using HNO_3. Following this, a solution of 1.49 g p-benzoquinone in 60 mL of absolute ethanol was added to the solution under vigorous stirring. Electrodeposition was performed using a CHI660 three-electrode setup at -0.1 V vs. Ag/AgCl for 5 minutes to create a layer of BiOI. Subsequently, 100 μL of a DMSO solution containing 0.23 M vanadium acetate oxide was applied onto the BiOI surface, followed by annealing at 450 °C (ramp rate = 2 °C/min) for 2 hours. Post-annealing, the photoanodes were immersed in a 1 M NaOH solution under stirring for 30 minutes to dissolve V_2O_5. The resulting $BiVO_4$ photoanodes were thoroughly rinsed with deionized water and dried using N_2.

Fabrication of Au/$BiVO_4$ photoanode. The gold sol was synthesized through the following procedure: Initially, 0.04 mmol of $HAuCl_4$ was dissolved in 50 mL of deionized water and heated to 95 °C. Subsequently, 5 mL of a 1% aqueous sodium citrate solution was introduced into the above solution under stirring and allowed to react for 15 min until the solution acquired a wine-red color. Finally, the prepared $BiVO_4$ photoanode was immersed in the gold sol for several hours, followed by thorough rinsing with deionized water.

Fabrication of Al_2O_3/Au/$BiVO_4$ photoanode. The prepared Au/$BiVO_4$ photoanodes were immersed in a solution of 0.3 mL aluminum sec-butoxide in 48.5 mL of isopropanol. This immersion was carried out at 60 °C for a duration of 30 min. Subsequently, the obtained photoanodes were thoroughly washed with deionized water and subjected to annealing at 200 °C for a period of 2 h.

Fabrication of $Co_4O_4/BiVO_4$, $Au@Co_4O_4(N)/BiVO_4$ photoanodes. The $BiVO_4$-based photoanodes underwent an immersion deposition process involving immersion in a CH_3OH solution containing 0.5 mM of Co_4O_4 for several hours. Subsequently, the photoanodes were sequentially rinsed with CH_3OH and deionized water, followed by blow-drying under a stream of N_2 gas.

Fabrication of $Au@Co_4O_4(A)/BiVO_4$ photoanode. The $Au@Co_4O_4(A)/BiVO_4$ photoanode was fabricated using an immersion method. Initially, a mixed solution of Co_4O_4 and Au NPs was created by combining 10 mL of Au sol with 10 mL of water containing 0.5 mM Co_4O_4. Subsequently, the $BiVO_4$ photoanode was immersed in the mixed solution and allowed to remain for several hours. Following immersion, the resulting photoanode was thoroughly rinsed with deionized water and subsequently dried under a stream of N_2 gas.

Fabrication of $Al_2O_3/Au@Co_4O_4(A)/BiVO_4$ photoanode. We conducted the atomic layer deposition (ALD) of Al_2O_3 using a sequential exposure method involving trimethylaluminum (TMA) and water. In each ALD cycle, the reaction chamber was maintained at 60 °C, with TMA exposure lasting for 3 s, followed by an exposure time of 40 s and a nitrogen purge time of 180 s. Water exposure took place over 30 s, followed by a nitrogen purge time of 180 s. By repeating this cycle five times, a uniform aluminum oxide layer was achieved, completely covering the surface of the $Au@Co_4O_4(A)/BiVO_4$ photoanode.

Photodeposition of PbO_2 on $Au/BiVO_4$ photoanode. In order to investigate the morphological alterations of Au nanoparticles (NPs) subsequent to PbO_2 deposition, we synthesized particles with a diameter of approximately 50 nm. The $Au/BiVO_4$ photoanode was prepared through the same impregnation method. Subsequently, the $Au/BiVO_4$ photoanode was immersed in an aqueous solution of $Pb(NO_3)_2$ (0.05 M), while a bare fluorine-doped tin oxide (FTO) counter electrode, electrically connected to the photoanode, was submerged in an aqueous $AgNO_3$ solution (0.05 M). The two electrolytes were connected via a proton exchange membrane. Finally, the $Au/BiVO_4$ photoanode was subjected to irradiation using a 300 W xenon lamp with an optical cut-off filter ($\lambda \geq 420$ nm) to facilitate hole-involved oxidative photo-deposition of PbO_2 on the $Au/BiVO_4$ photoanode and electron-involved reductive photo-deposition of Ag on the counter electrode.

FDTD simulation

The simulation employs a PML (perfect matching layer) as the boundary condition, defined within a cubic volume of 2000 nm × 2000 nm × 2000 nm, with a mesh size of 0.35 nm × 0.35 nm × 0.35 nm. Monochromatic linearly polarized light with a wavelength of 532 nm serves as the light source in the simulation calculations. The light source's polarization aligns with the y-axis, while its incidence occurs along the z-axis. The TFSF (total field scattered field) light source is configured with dimensions of 600 nm × 600 nm × 200 nm. The Au NPs are predominantly of size 20 nm, and the separation between the two Au NPs corresponds to the length of the Co_4O_4 molecule (1.6 nm).

Intensity modulated photocurrent spectroscopy (IMPS) measurement

IMPS measurements were carried out using a Zahner IMPS electrochemical workstation, controlled by a potentiostat (IM6ex, Zahner Company). A light-emitting diode (LED) was utilized for intensity-modulated light, enabling the superimposition of sinusoidal modulation (10%) on a direct current (dc) illumination level. The wavelength of the light source was 460 nm, with an average intensity of 10 W/m^2. Photocurrent responses over a range of frequencies (from 10 kHz to 0.1 Hz) were recorded at various potentials. The electrolyte employed was a 0.1 M phosphate buffer solution (PBS) with a pH of 7.

Characterization

X-ray Power Diffraction (XRD) patterns of the $BiVO_4$-based photoanodes were collected using a Bruker D8 QUEST powder diffractometer. The scan was conducted at a rate of 20°/min within the range of 20-80°. UV-visible (UV-vis) diffuse reflectance spectra were recorded on a JASCO V-650 UV-vis spectrophotometer equipped with an integrating sphere. Raman spectra were acquired using a commercial Raman spectrometer with a 532 nm diode-pumped solid-state laser. The morphology of the $BiVO_4$-based photoanodes was examined by scanning electron microscopy (SEM, Quanta 200 FEG, FEI) and high-resolution transmission electron microscopy (HRTEM, JEOL 2100). High angle annular dark field (HAADF) images were obtained using a Tecnai G2 F30 electron microscope. Compositional elements were analyzed using energy dispersive X-ray spectrometry (EDS) with data acquisition at 200 kV. Dynamic Light Scattering (DLS) measurements were performed using

equipment from nazo zs. X-ray photoelectron spectra (XPS) were measured on a Thermo Scientific ESCALAB 250 instrument with Al Kα radiation at 1486.6 eV, a spot size of 500 μm, and 150 W power to determine surface elements. Photoluminescence (PL) spectra were recorded on a Hitachi F-7000 spectrophotometer, and PL intensity was calculated by curve integration. Luminescence decay curves were obtained using a Lecroy Wave Runner 6100 digital oscilloscope (1 GHz) with a tunable laser as the excitation source (460 nm). PL decay profiles were acquired through time-correlated single photon counting at 25 °C, with detection wavelength set to 490 nm.

Photoelectrochemical (PEC) measurement

PEC experiments were conducted using a three-electrode electrochemical cell with a CHI 660E potentiostat instrument (Shanghai Chenhua Instrument Co., Ltd.) at a temperature of 25 °C. The three-electrode setup consisted of the as-prepared photoanode as the working electrode, a platinum tablet as the counter electrode, and an Ag/AgCl electrode as the reference electrode. Illumination of the photoanode was achieved using a Xe lamp (CEL-HXF300C) equipped with an AM 1.5G-filter, and the light intensity was adjusted and calibrated to 100 mW/cm^2. The light was directed onto the rear surface of the photoanode. Current-voltage (J-V) curves were obtained via linear sweep voltammetry with a scan rate of 20 mV/s. Electrochemical Impedance Spectroscopy (EIS) measurements were performed under irradiation at 0.6 V vs. RHE over a frequency range from 0.1 Hz to 100,000 Hz. Mott-Schottky measurements were conducted with a potential range from -0.8 V to -0.6 V vs. Ag/AgCl at a frequency of 1 kHz. Typically, a 0.1 M phosphate buffer solution (PBS) with a pH of 7 served as the electrolyte. The conversion between potentials relative to Ag/AgCl and the reversible hydrogen electrode (RHE) was achieved using the following equation:

$$E \text{ (vs. RHE)} = E \text{ (vs. Ag/AgCl)} + 0.0591 \text{ V} \times \text{pH} + E_{Ag/AgCl} \text{ (reference)}$$

The applied bias photon-to-current efficiency (ABPE) was calculated from the J-V curve, where J is the photocurrent density, V_{bias} is the applied bias, and P_{in} is the incident illumination power density (AM 1.5G, 100 mW/cm^2),

$$ABPE = \frac{J \times (1.23 - V_{bia})}{P_{in}} \times 100\%$$

The photocurrent density arising from PEC water oxidation can be described as:

$$J_{H_2O} = J_{abs} \times \eta_{sep} \times \eta_{trans}$$

where J_{abs} is the photocurrent density when the absorbed photons completely convert into the current, η_{sep} is the charge separation efficiency of the photogenerated holes that refer to the bulk recombination, and η_{trans} is the charge transfer efficiency of the surface reaching holes into the electrolyte. With Na_2SO_3 as a hole scavenger, the surface recombination is eliminated, η_{trans} = 1, and the photocurrent density can be described as:

$$J_{\mathrm{Na_2SO_3}} = J_{\mathrm{abs}} \times \eta_{\mathrm{sep}}$$

So the η_{trans} can be described as:

$$\eta_{trans} = \frac{J_{\mathrm{H_2O}}}{J_{\mathrm{Na_2SO_3}}}$$

Incident photon-to-current efficiency (IPCE) at various wavelengths was assessed using a 300 W Xe arc lamp, coupled with neutral density filters to simulate solar output. Monochromatic light was generated employing an Oriel Cornerstone 130 monochromator with a 10-nm bandpass, and the resulting output was quantified via a photodiode detector. IPCE measurements were conducted at 1.23 V vs. RHE in a 0.1 M phosphate buffer solution (PBS), utilizing the identical three-electrode configuration detailed earlier for photocurrent measurements.

$$IPCE = \frac{1240 \times J}{\lambda \times P_{in}} \times 100\%$$

where J is the photocurrent density, λ is the incident light wavelength, and P_{in} is the measured irradiance.

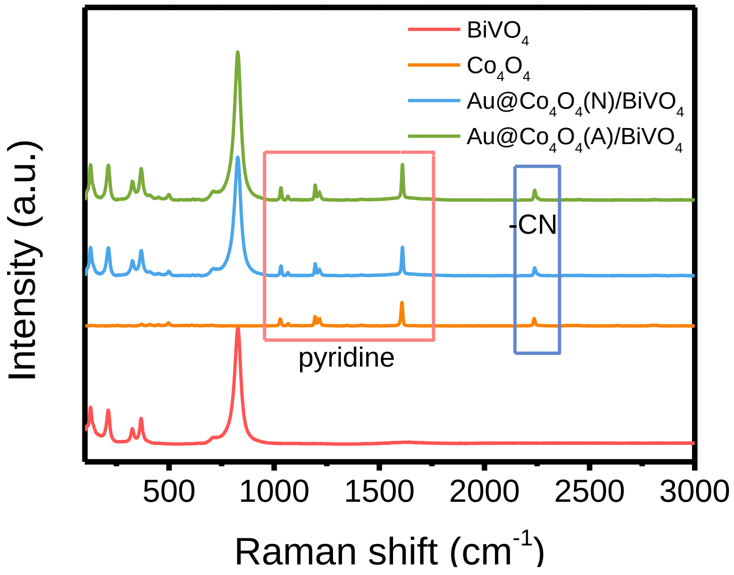

Figure S1. Raman spectra of Co_4O_4 molecules, $BiVO_4$, Au@Co_4O_4(N)/$BiVO_4$ and Au@Co_4O_4(A)/$BiVO_4$.

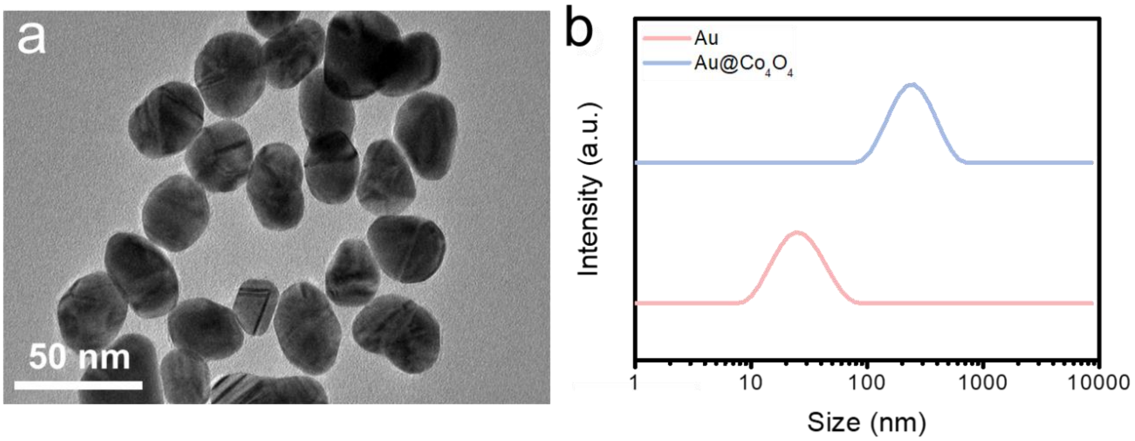

Figure S2. (a) TEM images of Au NPs. (b) Dynamic Light Scattering (DLS) of those compounds.

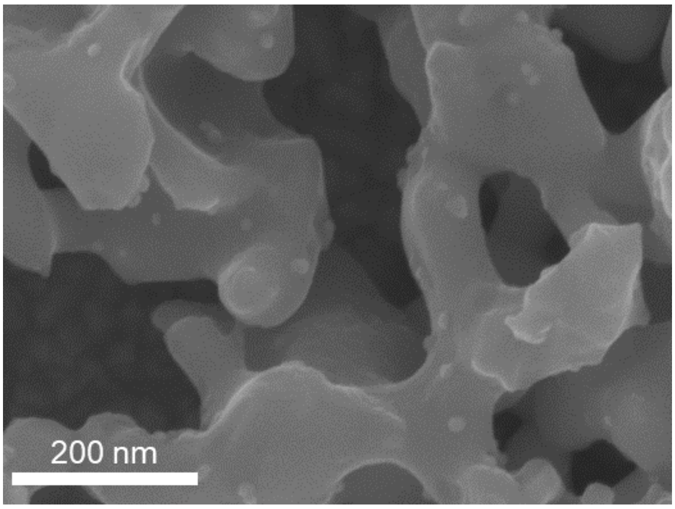

Figure S3. SEM image of Au/$BiVO_4$.

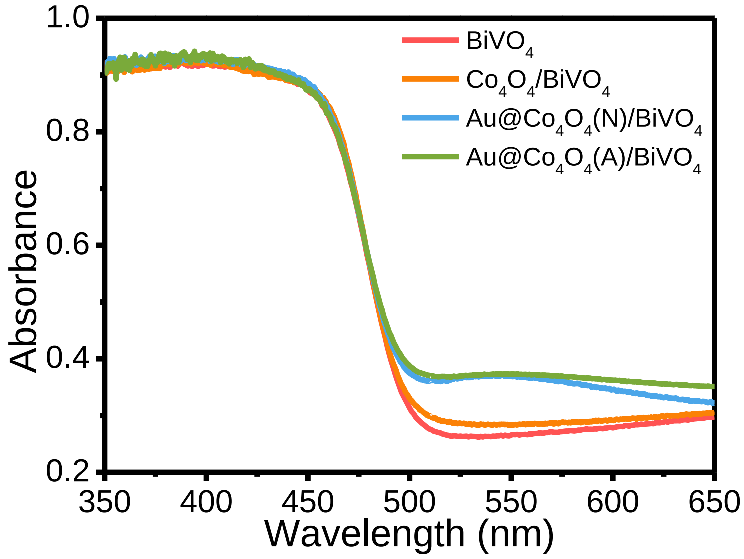

Figure S4. UV-vis spectra of $BiVO_4$-based photoanodes.

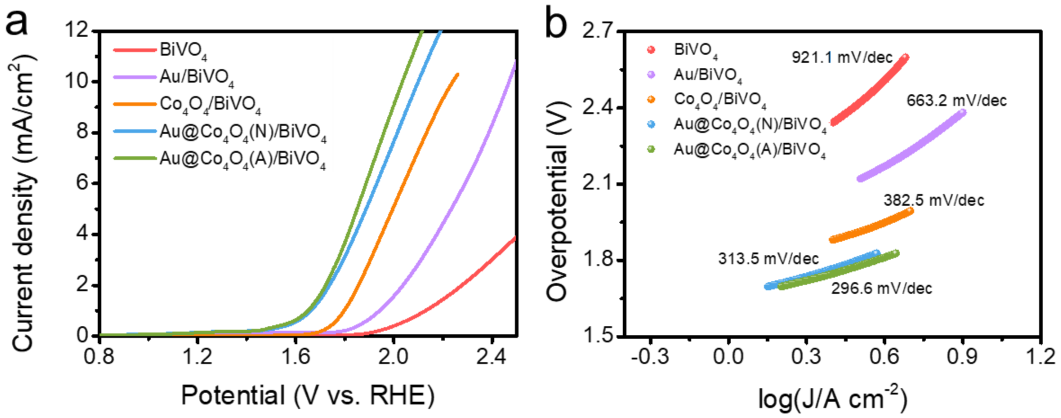

Figure S5. (a) Current-potential curves and (b) Tafel curves of the $BiVO_4$-based photoanodes.

As depicted in **Figure S5a**, the $BiVO_4$ electrode exhibits comparatively lower catalytic activity for water oxidation. However, upon the assembly of Au or Co_4O_4 molecules onto the $BiVO_4$ surface, notable enhancements in current and a negative shift in the onset potential are evident, indicating an improved catalytic activity for water oxidation. Intriguingly, both Au@Co_4O_4(N)/$BiVO_4$ and Au@Co_4O_4(A)/$BiVO_4$ exhibit further reductions in the onset potential, from 1.71 V_{RHE} to 1.48 V_{RHE}, in comparison to Co_4O_4/$BiVO_4$. Furtherly, the Tafel slope values for Au@Co_4O_4(N)/$BiVO_4$ and Au@Co_4O_4(A)/$BiVO_4$ photoanodes are 313.5 mV/dec and 296.9 mV/dec, respectively, which are smaller than those for Co_4O_4/$BiVO_4$ (382.5 mV/dec) and Au/$BiVO_4$ (663.2 mV/dec) (**Figure S5b**). Based on the above results, we can conclude that the synergistic enhancement in the kinetics of the water oxidation reaction facilitated by Au and Co_4O_4 molecules.

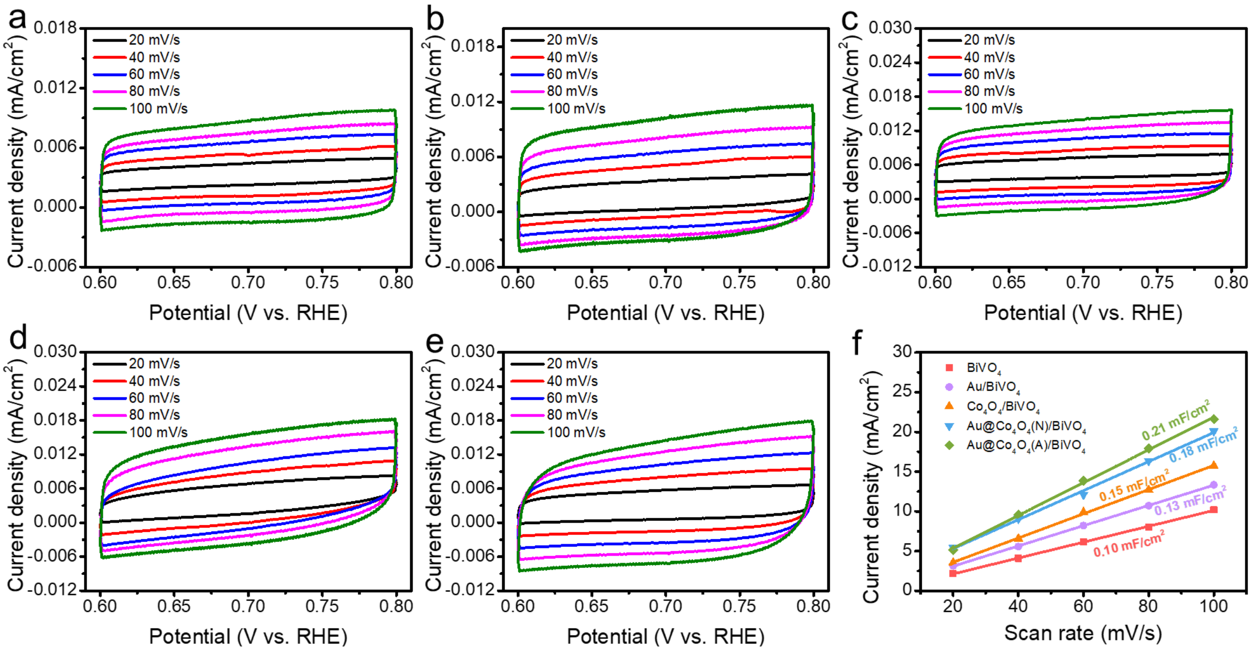

Figure S6. Cyclic voltammograms under dark conduction for (a) $BiVO_4$, (b) Au/$BiVO_4$, (c) Co_4O_4/$BiVO_4$, (d) Au@Co_4O_4(N)/$BiVO_4$ and (e) Au@Co_4O_4(A)/$BiVO_4$ (between 0.6 V_{RHE} to 0.8 V_{RHE}) at different scan rates (20, 40, 60, 80 and 100 mV/s) in a potential range without Faradaic processes. (f) Charging current densities recorded at 0.7 V_{RHE} at different scan rates.

The electrochemically active surface area (ECSA) serves as a valuable tool for assessing the water oxidation properties at the photoanode surface. ECSA was determined by calculating the double-layer capacitance (C_{dl}) of the electrode surface, which was derived from the slope of current densities with respect to scan rates in cyclic voltammetry (CV) curves (**Figure S6a-S6e**). It is noteworthy that both Au@Co_4O_4(N)/$BiVO_4$ and Au@Co_4O_4(A)/$BiVO_4$ exhibit significantly higher C_{dl} values, measuring 0.21 mF/cm^2 and 0.18 mF/cm^2, respectively, in comparison to Co_4O_4/$BiVO_4$ (0.15 mF/cm^2) and Au/$BiVO_4$ (0.13 mF/cm^2) (**Figure S6f**). This observation underscores the synergistic increase in ECSA resulting from the combination of Au and Co_4O_4 molecules.

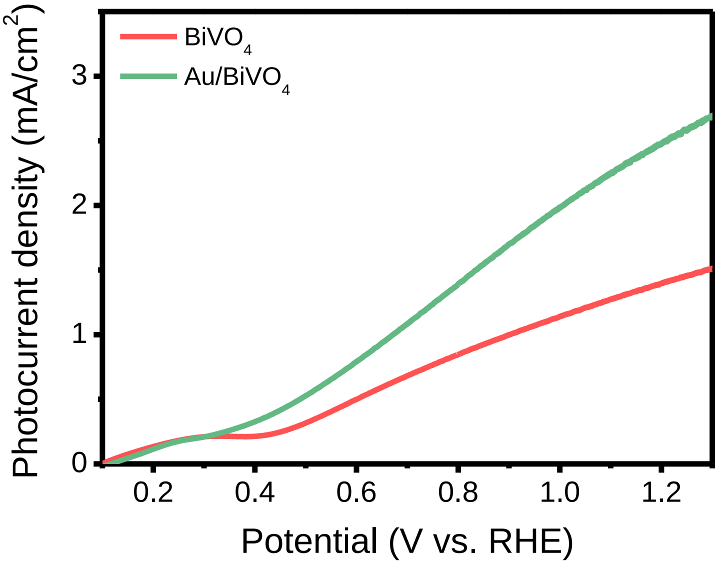

Figure S7. LSV curves of the $BiVO_4$ and $Au/BiVO_4$ photoanodes.

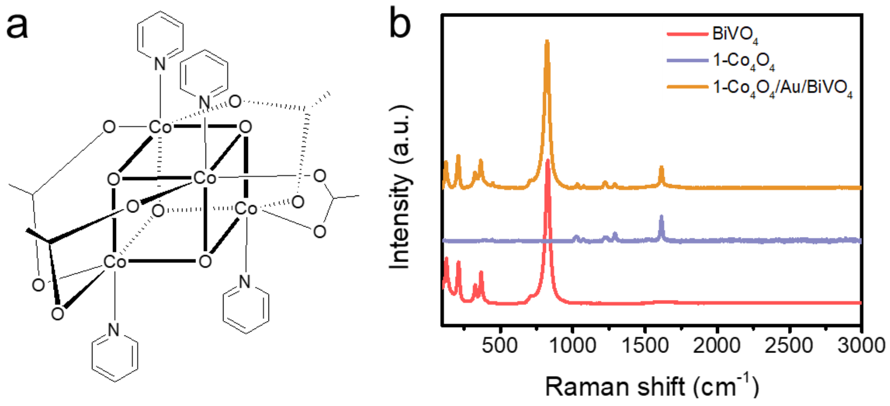

Figure S8. (a) The structure of 1-Co_4O_4 molecules. (b) Raman spectra of $BiVO_4$, 1-Co_4O_4 molecules and 1-Co_4O_4/Au/$BiVO_4$.

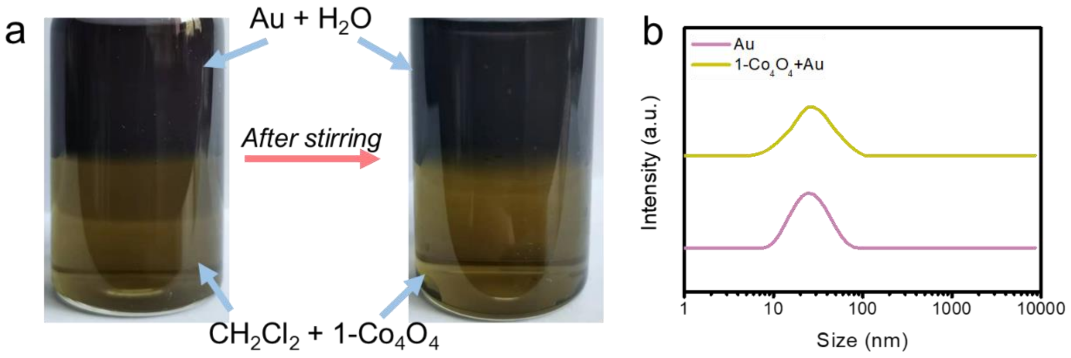

Figure S9. (a) The biphasic adsorption experiment of Au sol and 1-Co_4O_4 molecules. (b) Dynamic Light Scattering (DLS) of those compounds.

To verify the interaction between the CN- group and Au NPs, a controlled experiment was conducted for biphasic adsorption. As shown in **Figure S9a**. the upper layer solution is Au sol and the lower

layer solution is dichloromethane (CH_2Cl_2) solution containing 1-Co_4O_4 molecules. With the increase of the stirring time, the Au sol and CH_2Cl_2 solution contained 1-Co_4O_4 molecules still maintained the original layered state. From dynamic light scattering (DLS) measurements, the 1-Co_4O_4 molecules and Au NPs mixture presents a particle size distribution close to that of Au sol, further demonstrating the significant of roles of CN- group to bind the Au NPs (**Figure S9b**).

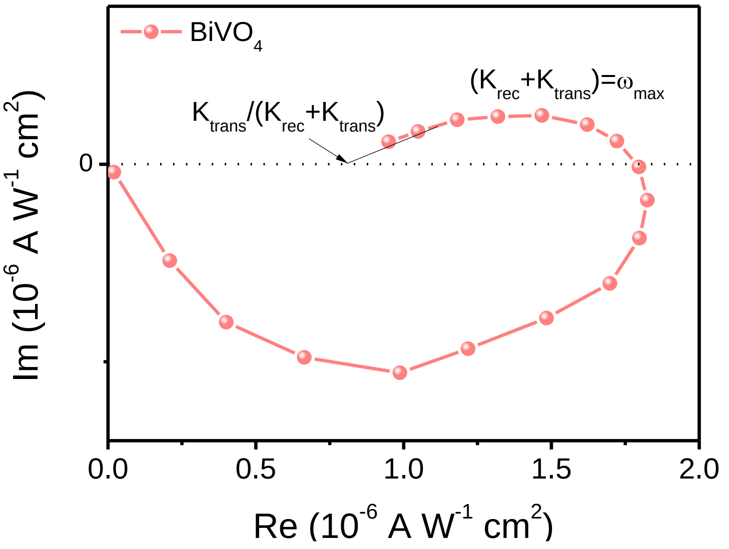

Figure S10. A calculated example of IMPS spectrum, illustrating the recombination (upper quadrant) semicircles.

Within an expansive frequency range, a discernible IMPS curve displays two prominent semicircles positioned in the first and fourth quadrants. As delineated earlier (**Figure S10**), the pseudo-first-order charge transfer and recombination rate constants, denoted as k_{trans} and k_{rec} respectively, are expressed in units of s^{-1}. The intercepts at high and low frequencies along the x-axis (where imaginary current equals zero) mirror the initial peak and the steady-state photocurrents encountered in a transient photocurrent plot. Remarkably, these constants adhere to the relationship ($k_{trans} + k_{rec} = 2\pi f_{max}$). Furthermore, the low-frequency intercept signifies the proportion of holes situated on the photoanode surface that undergo successful injection into the electrolyte. Evidently, this parameter encapsulates the essence of charge-transfer efficiency ($k_{trans}/(k_{trans} + k_{rec})$).

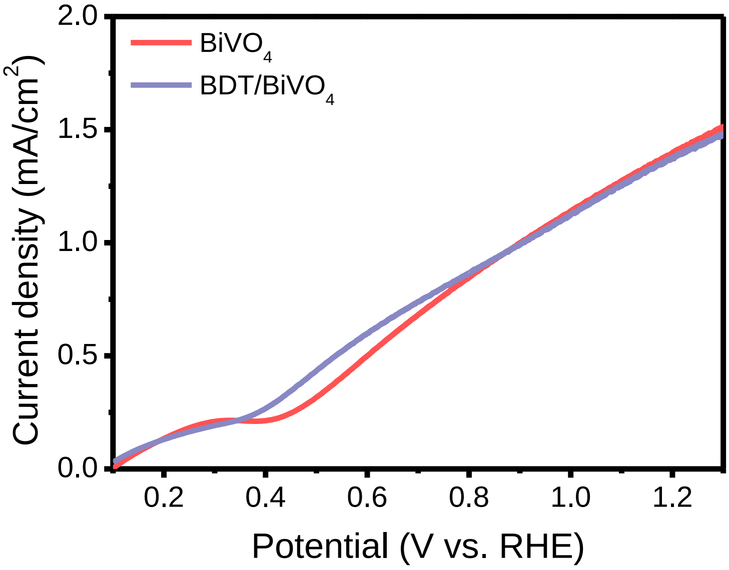

Figure S11. LSV curves of $BiVO_4$ with and without the treatment of 1,2-Benzenedithiol (BDT).

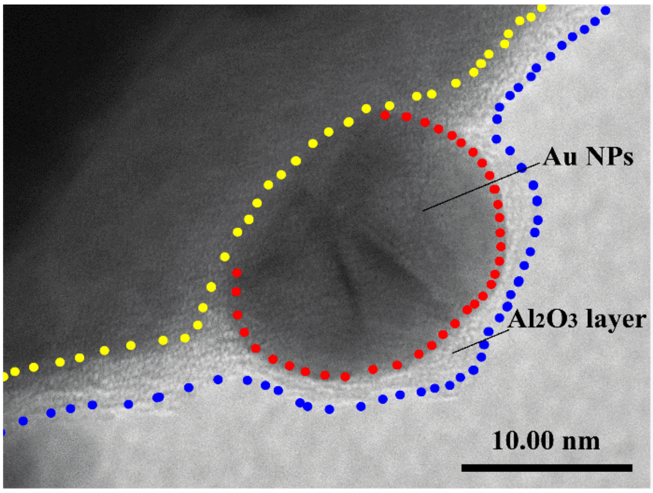

Figure S12. TEM image of Al_2O_3/Au/$BiVO_4$.

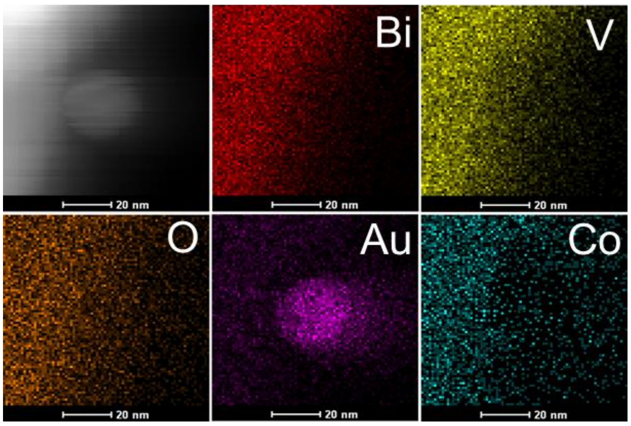

Figure S13. HAADF-STEM and EDS elemental mapping images of 1-Co_4O_4/Au/$BiVO_4$.

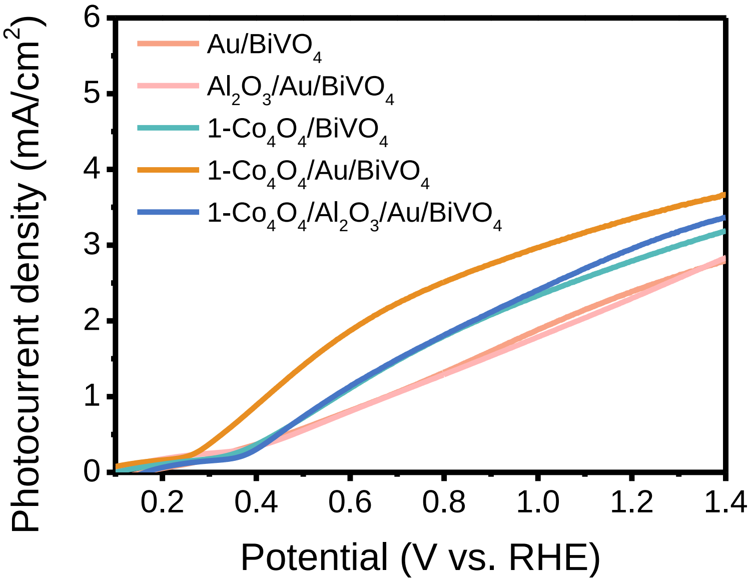

Figure S14. The LSV curves of $BiVO_4$-based photoanodes.

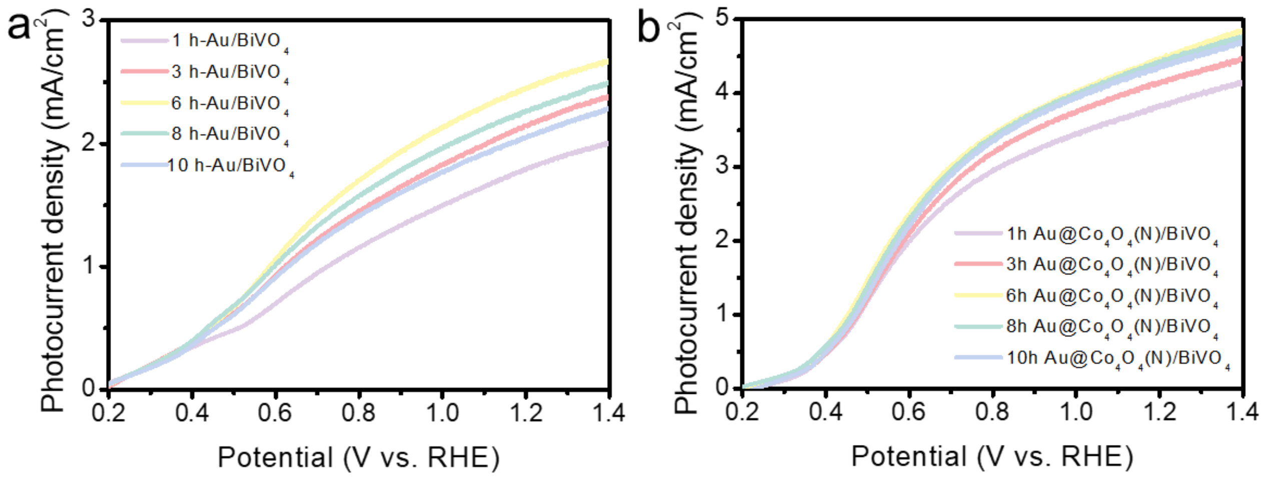

Figure S15. The LSV curves of (a) $Au/BiVO_4$ and (b) $Au@Co_4O_4(N)/BiVO_4$ photoanodes with different dipping time.

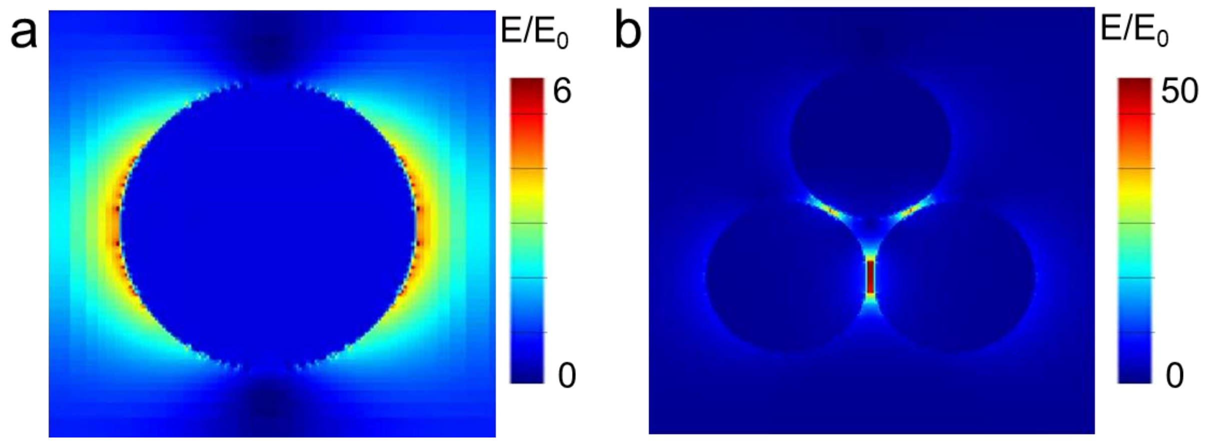

Figure S16. FDTD simulation results of near-feld enhancement for (a) Au NP and (b) Au@Co_4O_4 aggregates.

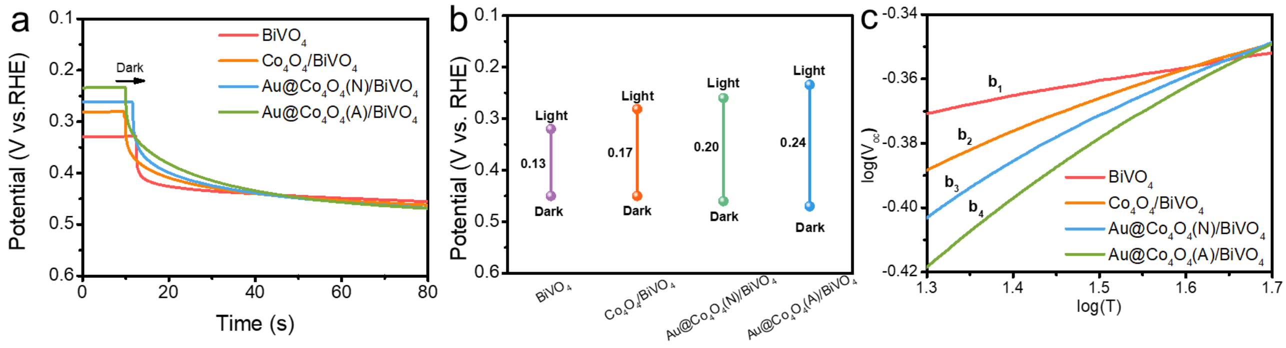

Figure S17. (a) The open-circuit potential decay curves of $BiVO_4$-based photoanodes. (b) Photovoltages obtained by open-circuit potential values and (c) log(V_{OC}) vs log t plots of $BiVO_4$-based photoanodes.

To discern the transport dynamics of photogenerated charges between the Co_4O_4 catalyst and the $BiVO_4$ photoanode, we calculated both the photovoltage and the surface recombination rate using open-circuit potential curves. Initial open-circuit potential measurements were performed under illumination, followed by data collection with the light source turned off (**Figure S17a**). The photovoltage was subsequently determined by contrasting the open-circuit potential values obtained under illuminated and dark conditions (**Figure S17b**). For evaluating the surface recombination rate, we employed the relationship: $V_{OC} = V_{light} \times t \times b$, where V_{OC} signifies the open-circuit potential at a specific time, V_{light} is the open-circuit potential under illumination, t represents the decay time, and b represents the recovery constant. Notably, a smaller value of recovery constant (b) implies a more rapid recombination rate for photo-generated charges. As depicted in **Figure S17c**, the sequence of recovery constants (b) among the photoanodes aligns with the expected order: Au@Co_4O_4(N)/$BiVO_4$ > Au@Co_4O_4(A)/$BiVO_4$ > Co_4O_4/$BiVO_4$ > $BiVO_4$, correspondingly reflecting an escalating trend of charges recombination.

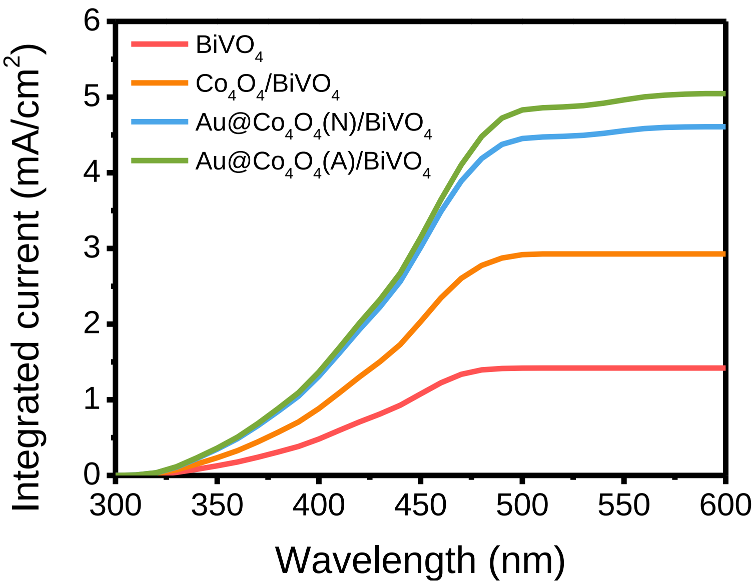

Figure S18. The calculated photocurrent of $BiVO_4$-based photoanodes by integrating IPCE at 1.23 V vs. RHE over the photon flux of AM 1.5G.

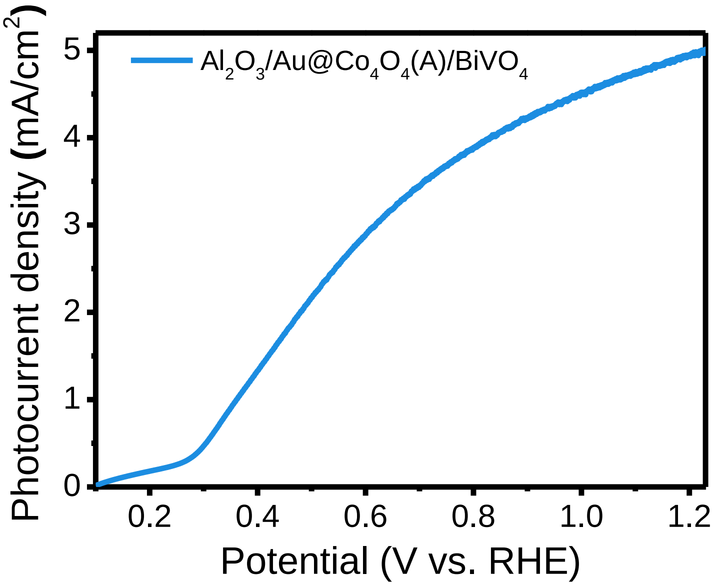

Figure S19. LSV curves of Al_2O_3/Au@Co_4O_4(A)/$BiVO_4$ photoanode.

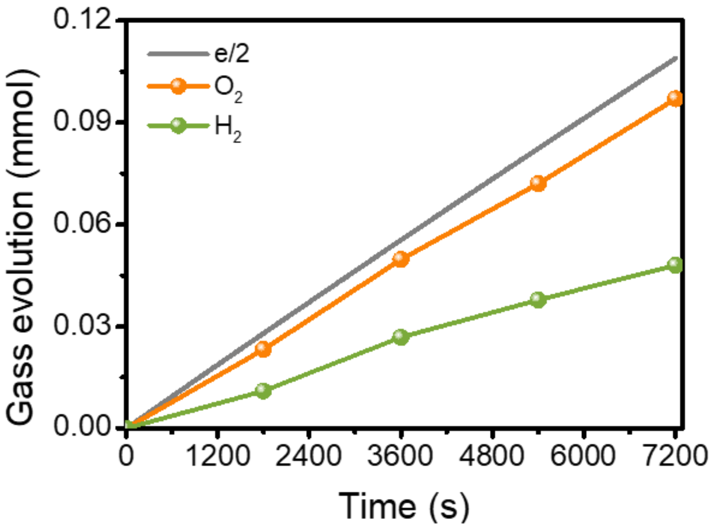

Figure S20. Oxygen and hydrogen evolution curves of the Al_2O_3/Au@Co_4O_4(A)/$BiVO_4$ photoanode at 0.7 V vs. RHE.

Table S1. The average lifetime of $BiVO_4$-based photoanodes.

Photoanodes	τ_1 (ns)	Proportion (P_1)	τ_2 (ns)	Proportion (P_2)	τ_{ave} (ns)
$BiVO_4$	0.06	72%	6.4	28%	1.8
Co_4O_4 /$BiVO_4$	0.05	70%	7.63	30%	2.3
Au@Co_4O_4(N)/$BiVO_4$	0.12	58%	9.33	42%	4.0
Au@Co_4O_4(A)/$BiVO_4$	0.1	64%	18.82	36%	6.8

Time-resolved photoluminescence (TRPL) measurements were conducted to gain deeper insights into the charge-transfer dynamics within the $BiVO_4$-based photoanodes. The intensity-weighted average exciton lifetime (τ_{ave}) was determined through the equation ($p_1\tau_1 + p_2\tau_2$), where p_1 and p_2 represent the fractional intensities, and τ_1 and τ_2 correspond to the lifetimes obtained from the PL decay analysis. Notably, the PL decay profile of Au@Co_4O_4(A)/$BiVO_4$ exhibited the longest lifetime (**Figure 6b**), characterized by a double exponential decay comprising a short-lived component (τ_1 = 0.1 ns, 64%) and a long-lived component (τ_2 = 18.82 ns, 36%) (**Table S1**). Calculations revealed an average PL lifetime of approximately 6.8 ns for Au@Co_4O_4(A)/$BiVO_4$, which notably surpasses that of other photoanode samples.

Table S2. PEC water oxidation performances of recently reported molecule/semiconductor hybrid photoanodes.

Photoanodes	Electrolyte	Photocurrent density (mA/cm^2 @ 1.23 V_{RHE})	References
(Co_4O_4)1h/$BiVO_4$	0.5M borate buffer (pH 9.3)	5.00	[1]
$CoPO_3$/pGO/LDH/$BiVO_4$	0.1 M PBS (pH 9.0)	4.45	[2]
cobaloxime/Ni-OEC/BVO	1 M KBi (pH 9)	5.10	[3]
Co@CB[5]/$BiVO_4$	1 M borate buffer (pH 9)	4.80	[4]
CuTCPP/GO/$BiVO_4$	0.5 M borate buffer (pH 9)	5.00	[5]
poly-1/Vpa/Al_2O_3/$BiVO_4$	0.1 M PBS (pH 7.0)	2.80	[6]
Co2/$BiVO_4$	0.1 M PBS (pH 7.0)	4.27	[7]
Co–cubane/TiO_2/$BiVO_4$	0.1 M PBS (pH 7.0)	4.60	[8]
Cobim/BVO-20	0.5 M Na_2SO_4 (pH 7)	3.10	[9]
Ni_4O_4/$BiVO_4$	0.2 M PBS (pH 7)	3.90	[10]
**1**/FTA/$BiVO_4$	0.5 M borate buffer (pH 9)	5.50	[11]
$BiVO_4$/$NdCo_3$	1 M borate buffer (pH 9)	2.25	[12]
Au@Co_4O_4(A)/$BiVO_4$	0.1 M PBS (pH 7.0)	5.06	This work

References

[1] Y. Wang, F. Li, X. Zhou, F. Yu, J. Du, L. Bai, L. Sun, *Angew Chem Int Ed.* **2017**, *56*, 6911-6915.

[2] S. Ye, C. M. Ding, R. T. Chen, F. T. Fan, P. Fu, H. Yin, X. L. Wang, Z. L. Wang, P. W. Du, C. Li, *J. Am. Chem. Soc.* **2018**, *140*, 3250-3256.

[3] T. T. Wang, H. Y. Cao, J. B. Wu, M. G. Haghighi, R. Sedghi, P. W. Du, *J. Phys. Chem. C* **2022**. *126*, 11042-11050.

[4] F. Li, H. Yang, Q. Zhuo, D. Zhou, X. Wu, P. Zhang, Z. Yao, L. Sun, *Angew Chem Int Ed.* **2021**, *60*, 1976-1985.

[5] C. Xu, W. Sun, Y. Dong, C. Dong, Q. Hu, B. Ma and Y. Ding, *J. Mater. Chem. A* **2020**, *8,* 4062-4072.

[6] W. Jiang, X. Yang, F. Li, Q. Zhang, S. Li, H. Tong, Y. Jiang and L. Xia, *Chem. Commun.* **2019**, *55*, 1414-1417.

[7] Y. D. Liu, Y. Jiang, F. Li, F. S. Yu, W. C. Jiang, L. X. Xia, *J. Mater. Chem. A* **2018**, *6*, 10761-10768.

[8] W. C. Jiang, Y. Jiang, J. Tong, Q. Zhang, S. Y. Li, H. L. Tong, L. X. Xia, *Rsc Advances* **2018**, *8*, 41439-41444.

[9] W. Zhang, R. Li, X. Zhao, Z. Chen, A. W. K. Law, K. Zhou, *ChemSusChem* **2018**, *11*, 2710-2716.

[10] B. Gao, T. Wang, X. L. Fan, H. Gong, P. Li, Y. Y. Feng, X. L. Huang, J. P. He, J. H. Ye, *J. Mater. Chem. A* **2019**, *7*, 278-288.

[11] X. H. Cao, C. J. Xu, X. M. Liang, J. R. Ma, M. E. Yue, Y. Ding, *Appl. Catal., B* **2020**, *260*, 118136.

[12] G. D. Gao, R. Chen, Q. J. Wang, D. W. F. Cheung, J. Zhao, J. S. Luo, *Acs Applied Energy Materials* **2023**, *6*, 4027-4034.
